# Association of Dysphagia Severity with Nutritional Status and Muscle Function in Outpatients with Multiple Sclerosis: A Cross-Sectional Study

**DOI:** 10.3390/medicina62071271

**Published:** 2026-06-30

**Authors:** Nezihe Otay Lule, Hakan Polat, Yasemin Ekmekyapar Firat

**Affiliations:** 1 Nutrition and Dietetics Department, Faculty of Health Sciences, Gaziantep University, 27310 Gaziantep, Türkiye; 2 Physiotherapy and Rehabilitation Department, Faculty of Health Sciences, SANKO University, 27310 Gaziantep, Türkiye; fzthakanpolat@gmail.com; 3 Department of Neurology, Faculty of Medicine, SANKO University, 27310 Gaziantep, Türkiye; yaseminekmekyapar@gmail.com

**Keywords:** multiple sclerosis, dysphagia, malnutrition, sarcopenia, GLIM criteria, EWGSOP2, nutritional screening, MUST, DYMUS, EAT-10

## Abstract

*Background/Objectives*: Dysphagia may adversely affect nutritional status in patients with Multiple Sclerosis (MS). This study aimed to investigate the associations between dysphagia severity and (i) nutritional status, assessed by the Malnutrition Universal Screening Tool (MUST) and Global Leadership Initiative on Malnutrition (GLIM) criteria, and (ii) secondary sarcopenia indicators according to the European Working Group on Sarcopenia in Older People-2 (EWGSOP2) framework. *Materials and Methods*: This cross-sectional study enrolled 32 consecutive adult outpatients with confirmed MS and self-reported dysphagia (DYMUS ≥ 1). Dysphagia severity was evaluated using the Dysphagia in Multiple Sclerosis (DYMUS) questionnaire, the Eating Assessment Tool-10 (EAT-10), and the Yale Swallow Protocol. Nutritional assessment included MUST screening and GLIM-based malnutrition diagnosis. Muscle function was evaluated via handgrip strength, calf circumference, and 4-metre gait speed. *Results*: GLIM-defined malnutrition was identified in 12 (37.5%) patients. Dysphagia severity was significantly associated with MUST score (ρ = 0.596, *p* < 0.001) and the presence of GLIM-defined malnutrition (median DYMUS 6.5 vs. 4.0; *p* = 0.012). In exploratory logistic regression, higher DYMUS scores were associated with GLIM-defined malnutrition. Conversely, no significant associations were found between dysphagia severity and handgrip strength, calf circumference, or sarcopenia classification (*p* > 0.30 for all). The categorical severe-sarcopenia rate was not considered reliably interpretable because of a pronounced gait speed floor effect. *Conclusions*: In ambulatory MS patients with dysphagia, dysphagia severity was associated with nutritional risk indicators and GLIM-defined malnutrition, but not with the primary muscle strength and mass indicators evaluated. Because MUST and GLIM reflect composite nutritional risk rather than confirmed protein–energy deficiency, these findings should be regarded as exploratory and hypothesis-generating. The present data did not permit a reliable estimate of sarcopenia prevalence because of a pronounced gait speed floor effect and the absence of body composition measurement. As a preliminary practical consideration, these findings may support combined dysphagia and nutritional screening in multidisciplinary MS outpatient care, pending confirmation in larger prospective cohorts.

## 1. Introduction

Multiple sclerosis (MS) is a chronic, immune-mediated, demyelinating disease of the central nervous system, characterised by progressive neurological disability and a wide spectrum of physical and cognitive impairments [1]. MS affects approximately 2.8 million individuals worldwide and represents the leading non-traumatic cause of neurological disability in young adults [2]. Among the diverse neurological manifestations of MS, dysphagia—defined as impairment in the safe or efficient transport of a food or liquid bolus from the mouth to the stomach—has received comparatively little clinical attention, despite its substantial impact on patient outcomes [3].

The pooled prevalence of dysphagia in MS has been estimated at approximately 45% (95% CI: 40.4–49.2%) across patient populations, with significant variation related to disease subtype, disability level, and assessment method [4]. The consequences of dysphagia extend beyond impaired swallowing mechanics: inadequate oral intake, aspiration risk, recurrent respiratory infections, and deterioration in health-related quality of life are all well-documented sequelae [5,6].

From a nutritional perspective, dysphagia compromises both the efficiency and safety of food intake. Restricted dietary choices, reduced meal volumes, and fear of eating collectively contribute to inadequate energy and protein ingestion, thereby elevating the risk of disease-related malnutrition [7]. Malnutrition in MS is a multifactorial phenomenon driven by dysphagia, elevated inflammatory burden, physical inactivity, and autonomic dysfunction, and is associated with accelerated functional decline and increased healthcare utilisation [8]. Despite this, nutritional assessment is not yet systematically embedded in MS clinical pathways, particularly in the outpatient setting.

Malnutrition-related protein-energy deficit creates conditions conducive to the development of secondary sarcopenia—progressive loss of skeletal muscle mass, strength, and physical performance attributable to disease rather than primary ageing [9]. In MS, chronic neuroinflammation, neuromuscular dysfunction, mobility restrictions, and reduced dietary intake collectively represent plausible pathophysiological drivers of secondary sarcopenia as conceptualised within the EWGSOP2 framework [10]. However, while individual components of this triad—dysphagia, malnutrition, and muscle dysfunction—have been studied separately, studies that simultaneously and systematically evaluate all three in ambulatory MS patients remain scarce.

The Global Leadership Initiative on Malnutrition (GLIM) criteria, introduced in 2019, provide a consensus-based, language-independent framework for the diagnosis of malnutrition in clinical settings, requiring at least one phenotypic and one aetiological criterion [11]. Crucially, in patients with dysphagia, reduced food intake constitutes an aetiological criterion, and low body mass index (BMI) or reduced lean tissue mass constitutes phenotypic criteria—rendering the GLIM framework particularly appropriate for this patient group. Similarly, the MUST represents a validated, age-independent screening tool applicable across care settings [12].

To address the gap in the existing literature, this study aimed to (i) evaluate the association between dysphagia severity and nutritional status, assessed by MUST and GLIM criteria, and (ii) investigate whether dysphagia severity is associated with secondary sarcopenia indicators—handgrip strength, calf circumference, and gait speed—in ambulatory MS patients with dysphagia. We hypothesised that greater dysphagia severity would be associated with worse nutritional status (H1) and lower muscle function parameters (H2) in this population.

## 2. Materials and Methods

### 2.1. Study Design and Setting

This was a cross-sectional, descriptive-analytical study conducted at the Neurological Rehabilitation Unit of SANKO University Hospital, Gaziantep, Türkiye. Participants were enrolled consecutively over a three-month period, from 22 January 2026 to 8 May 2026, following ethics committee approval (21 January 2026). The study was conducted in accordance with the Declaration of Helsinki, and informed written consent was obtained from all participants prior to enrolment.

### 2.2. Participants

**Figure 1:**
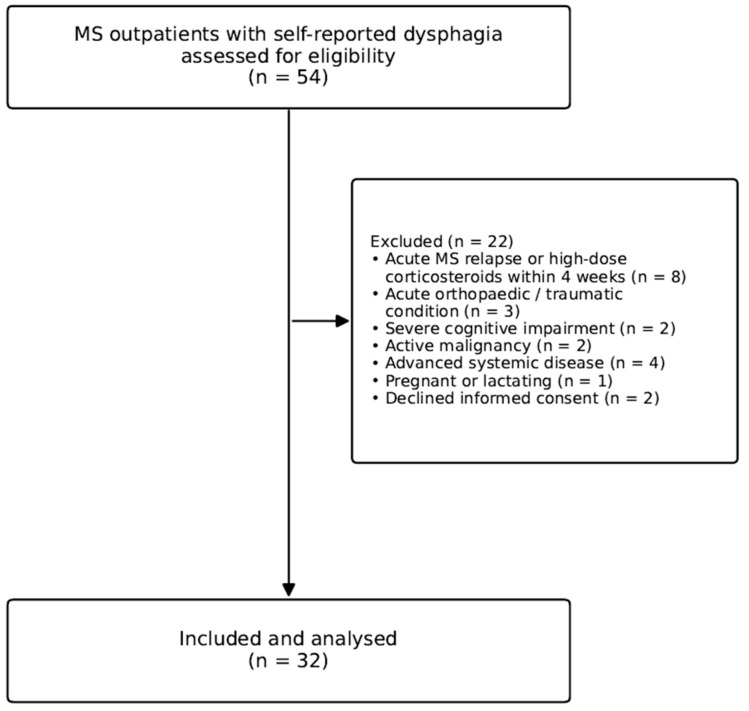
Participant flow diagram.

Eligible participants were adults (≥18 years) with a confirmed MS diagnosis according to the McDonald 2017 revised criteria and evidence of dysphagia, defined as a DYMUS score ≥ 1. Inclusion required availability of EDSS score in clinical records and sufficient cognitive and physical capacity to complete the study instruments. Patients were excluded if they had experienced an acute MS relapse or received high-dose corticosteroids within the preceding four weeks; had acute orthopaedic or traumatic conditions precluding assessment; had severe cognitive impairment precluding comprehension of instructions; were pregnant or lactating; had active malignancy or advanced systemic disease; or had any cervical pathology. Consecutive sampling was employed. During the study period, 54 MS outpatients with self-reported dysphagia were assessed for eligibility; 22 were excluded (acute MS relapse or high-dose corticosteroids within four weeks, *n* = 8; acute orthopaedic or traumatic condition, *n* = 3; severe cognitive impairment, *n* = 2; active malignancy, *n* = 2; advanced systemic disease, *n* = 4; pregnancy or lactation, *n* = 1; declined consent, *n* = 2), resulting in 32 included participants (Figure 1).

### 2.3. Sociodemographic and Clinical Data

Data were collected via structured face-to-face interview and clinical record review. Variables recorded included age, sex, marital status, MS clinical subtype (RRMS/SPMS/other), disease duration, number of relapses in the past two years, and EDSS score. Regular physical activity was defined as ≥150 min per week of moderate-intensity exercise over the preceding three months.

### 2.4. Dysphagia Assessment

Dysphagia was assessed using three complementary tools. The Eating Assessment Tool-10 (EAT-10) is a 10-item patient-reported outcome measure scoring dysphagia severity from 0 to 40; a score ≥ 3 indicates clinically significant dysphagia risk [13]. The Turkish validity and reliability study of EAT-10 was conducted by Demir et al. [14]. The Dysphagia in Multiple Sclerosis (DYMUS) questionnaire is an MS-specific, 10-item, yes/no self-report instrument assessing dysphagia for solids and liquids; scores range from 0 to 10, with higher scores reflecting greater dysphagia severity [15]. The Turkish validity and reliability of the DYMUS was established by Arslan et al. [16]. The Yale Swallow Protocol (YSP) was used as a bedside aspiration screening tool. Participants were asked to drink approximately 90 mL of water uninterruptedly; coughing, choking, voice change, or dyspnoea during or after the test constituted a positive (aspiration risk) result [17]. The YSP was used solely as a bedside screen for aspiration risk and does not provide a graded measure of dysphagia severity; accordingly, it was not used as a continuous predictor in the primary analyses.

### 2.5. Nutritional Assessment

The Malnutrition Universal Screening Tool (MUST) was used for nutritional risk screening [18]. MUST scores 0, 1, and ≥2 correspond to low, medium, and high malnutrition risk, respectively. The Turkish adaptation was validated by Sümer et al. [19]. Malnutrition diagnosis was established according to GLIM criteria [11], which require at least one phenotypic criterion (unintentional weight loss > 5% in six months, or >10% beyond six months; low BMI < 20 kg/m^2^ for patients aged <70 years; or reduced lean tissue mass assessed by calf circumference) and at least one aetiological criterion (reduced food intake or assimilation attributed to dysphagia; or inflammatory burden of chronic MS). Malnutrition severity was graded as moderate or severe based on phenotypic criteria thresholds. The MUST was used as the mandatory pre-screening tool per the GLIM stepwise approach.

Anthropometric measurements were performed according to standard protocols. Height was measured in the Frankfort plane using a stadiometer. Body weight was measured using a calibrated digital scale (precision 0.1 kg). BMI was calculated as weight (kg) divided by the square of height (m^2^). Calf circumference was measured at the widest point of the right calf with a non-elastic tape while the patient was seated with the knee at 90° [20].

### 2.6. Muscle Function Assessment (EWGSOP2 Framework)

Given the chronic inflammatory and neuromuscular pathophysiology of MS, muscle function parameters were evaluated within the EWGSOP2 secondary sarcopenia framework [10]. Handgrip strength was measured using a Jamar hydraulic hand dynamometer with the patient seated, elbow at 90° flexion, and wrist in neutral position. Three maximal attempts were performed with 30 s rest intervals; the highest value was recorded. Turkish population-specific cut-off values were applied: low muscle strength was defined as <32 kg in men and <22 kg in women [21]. Calf circumference < 33 cm in either sex served as a proxy for reduced muscle mass, consistent with GLIM recommendations in the absence of body composition analysis equipment [11]. Calf circumference was obtained from a single standardised measurement and entered unchanged into three frameworks (descriptive anthropometry, the GLIM phenotypic muscle-mass criterion, and the EWGSOP2 muscle-mass component); these appearances derive from one measurement and were not treated as independent evidence, and GLIM- and EWGSOP2-based endpoints are not presented as mutually confirmatory. Gait speed was assessed using the 4-metre walk test (4MWT) at habitual pace; a speed <0.8 m/s indicated low physical performance [10]. Sarcopenia classification followed the EWGSOP2 stepwise algorithm: possible sarcopenia (low muscle strength only), confirmed sarcopenia (low muscle strength plus low muscle mass), and severe sarcopenia (all three criteria met).

### 2.7. Power Analysis and Sample Size

Sample size was calculated a priori based on the primary objective of detecting a correlation between dysphagia severity and nutritional status. Using a two-tailed α = 0.05, power = 0.80, and an anticipated moderate-to-strong effect size (r = 0.50), a minimum of 29 participants was required. To account for potential missing data and the inclusion of confounding variables in secondary analyses, the target sample was extended to 32 consecutively enrolled patients.

### 2.8. Statistical Analysis

Data were analysed using IBM SPSS Statistics version 25.0 (IBM Corp., Armonk, NY, USA) and Python version 3.12 (Python Software Foundation, Wilmington, DE, USA), using the SciPy (version 1.16.0) and statsmodels (version 0.14.5) libraries. Normality was assessed using the Shapiro–Wilk test (appropriate for *n* < 50). Normally distributed continuous variables are presented as mean ± standard deviation (SD); non-normally distributed variables are presented as median and interquartile range (IQR); categorical variables are presented as frequency and percentage. Spearman’s rank correlation coefficient (ρ) was used to assess associations between dysphagia severity and continuous nutritional and muscle function variables. Between-group comparisons were performed using the Mann–Whitney U test (two groups) and the Kruskal–Wallis test with post hoc Dunn correction (three groups). Fisher’s exact test was applied for small-cell categorical comparisons. An exploratory binary logistic regression was conducted with GLIM-defined malnutrition as the dependent variable, with DYMUS score as the primary predictor; EDSS was included as a covariate in a secondary model. Linearity of the logit for the continuous predictor (DYMUS) was assessed using the Box–Tidwell test prior to interpretation of the logistic regression. Because reduced intake attributable to dysphagia is an aetiologic GLIM criterion, two sensitivity analyses were performed to assess the robustness of the DYMUS–malnutrition association to this overlap: GLIM was re-derived after excluding the dysphagia-related reduced-intake aetiologic criterion, and malnutrition was redefined using phenotypic criteria alone (≥1 phenotypic criterion, irrespective of aetiology), each modelled with DYMUS as the predictor. Given the small event count (*n* = 12 malnutrition cases), results are presented as hypothesis-generating with explicit acknowledgement of the limited events-per-variable ratio (EPV). No formal adjustment for multiple comparisons (e.g., Bonferroni or Benjamini–Hochberg correction) was applied to the correlation analyses given their exploratory intent; accordingly, the correlation analyses reported herein should be interpreted as hypothesis-generating and are subject to an increased family-wise type I error risk. Statistical significance was set at *p* < 0.05 (two-tailed). No data were missing for the variables included in the analyses; all analyses were performed on the complete sample (*n* = 32, complete-case analysis), and no imputation was required.

## 3. Results

### 3.1. Participant Characteristics

Thirty-two patients met the inclusion criteria and were enrolled (Figure 1). The sociodemographic, clinical, anthropometric, and assessment data are summarised in Table 1. The sample had a mean age of 40.3 ± 8.4 years and comprised predominantly female patients (81.2%). SPMS was the more common disease subtype (59.4%). The median EDSS score was 4.0 (IQR 3.5–4.0), indicating moderate disability. Median disease duration was 11.5 years (IQR 7.0–15.0). All participants exhibited at least mild dysphagia per DYMUS scoring; 43.8% were classified as moderate (DYMUS 4–6) and 25.0% as severe (DYMUS 7–10). Clinically significant dysphagia risk (EAT-10 ≥ 3) was present in 30 patients (93.8%). A positive Yale Swallow Protocol result (aspiration risk) was identified in five patients (15.6%). GLIM-defined malnutrition was present in 12 patients (37.5%), of whom nine (75.0% of malnourished) had severe malnutrition. Severe sarcopenia was the most common sarcopenia category, affecting 15 patients (46.9%). However, this figure is likely inflated by a pronounced floor effect in gait speed and should be interpreted as non-informative for sarcopenia burden.

### 3.2. Hypothesis 1: Dysphagia Severity and Nutritional Status

Spearman’s correlation analyses revealed statistically significant, moderate-to-strong positive associations between dysphagia severity and nutritional risk (Table 2). DYMUS score was significantly correlated with MUST score (ρ = 0.596, *p* < 0.001), as was EAT-10 (ρ = 0.518, *p* = 0.002). Neither DYMUS nor EAT-10 showed statistically significant associations with BMI or calf circumference (all *p* > 0.18), suggesting that anthropometric indices per se did not track closely with dysphagia severity in this sample.

Patients with GLIM-defined malnutrition demonstrated significantly higher DYMUS and EAT-10 scores and higher MUST scores than those without malnutrition, along with markedly lower BMI (Table 3). Calf circumference did not differ significantly between groups (*p* = 0.126). In an exploratory three-group comparison, DYMUS scores differed across MUST risk categories (H = 10.384, *p* = 0.006), with median DYMUS scores of 4.0, 4.0, and 7.0 in the low, medium, and high risk groups, respectively, and a parallel pattern for EAT-10 (H = 6.255, *p* = 0.044). However, the medium-risk category contained only 2 patients, rendering this three-group analysis statistically unreliable; it should therefore be regarded as exploratory and interpreted with caution. With only 5 aspiration-positive cases, the Yale Swallow Protocol comparison was underpowered; the absence of a significant difference between malnutrition groups (Fisher’s exact *p* = 0.338) is therefore non-informative rather than a true null finding.

### 3.3. Hypothesis 2: Dysphagia Severity and Muscle Function

Correlations between dysphagia severity and the primary EWGSOP2 muscle function parameters—handgrip strength and calf circumference—were statistically non-significant and weak in magnitude (Table 4). DYMUS score showed a negligible correlation with handgrip strength (ρ = 0.066, *p* = 0.720), as did EAT-10 (ρ = −0.021, *p* = 0.908). Similarly, neither measure of dysphagia severity correlated significantly with calf circumference.

In contrast, both DYMUS and EAT-10 showed statistically significant, moderate negative correlations with 4-metre gait speed as a continuous variable (DYMUS: ρ = −0.495, *p* = 0.004; EAT-10: ρ = −0.525, *p* = 0.002), indicating that more severe dysphagia was associated with slower gait. However, this finding warrants cautious interpretation: 30 of 32 participants (93.8%) were already classified as having low gait speed (<0.8 m/s) by EWGSOP2 thresholds, reflecting a pronounced floor effect in this outcome. Group comparisons confirmed the absence of a statistically significant association between dysphagia severity and sarcopenia classification: Kruskal–Wallis testing yielded H = 0.081 (*p* = 0.960) for DYMUS and H = 2.032 (*p* = 0.362) for EAT-10 across sarcopenia categories. Nor did handgrip strength category or calf circumference category differentiate dysphagia severity (all *p* > 0.30).

### 3.4. Exploratory Logistic Regression: Dysphagia Severity as a Predictor of Malnutrition

In the unadjusted model, each one-unit increase in DYMUS score was associated with significantly higher odds of GLIM-defined malnutrition (OR = 1.707; 95% CI: 1.138–2.560; *p* = 0.010). When EDSS was included as a covariate (exploratory model, EPV = 6), the association of DYMUS with malnutrition remained after exploratory adjustment for EDSS and was not substantially attenuated (OR = 1.722; 95% CI: 1.144–2.591; *p* = 0.009), while EDSS did not contribute independently (OR = 1.268; 95% CI: 0.427–3.769; *p* = 0.669) (Table 5). These results indicate that the association between dysphagia severity and malnutrition is not substantially confounded by overall neurological disability in this sample. However, with 12 events and an events-per-variable ratio of 6, this adjusted model is statistically unstable, the confidence intervals are wide, and the estimates should be regarded as hypothesis-generating pending confirmation in adequately powered cohorts. The Box–Tidwell test showed no evidence of departure from linearity of the logit for DYMUS (interaction term *p* = 0.157).

In the first sensitivity analysis, the dysphagia-related reduced-intake aetiologic criterion was met in 2 of 32 patients (6.3%), both of whom also satisfied a phenotypic criterion and the chronic-MS inflammatory aetiologic criterion; excluding this aetiologic route retained all 12 malnutrition cases and left the DYMUS odds ratio unchanged (OR = 1.707; 95% CI: 1.138–2.560). In the second analysis, 26 of 32 patients (81.3%) met ≥ 1 phenotypic criterion, and DYMUS score was not associated with this phenotype-only outcome (OR = 0.943; 95% CI: 0.656–1.354; *p* = 0.749).

### 3.5. Additional Analyses: MS Subtype and Muscle Function

Handgrip strength differed significantly between MS subtypes: median grip strength was 24.0 kg in RRMS patients versus 20.0 kg in SPMS patients (Mann–Whitney U = 210; *p* < 0.001). This difference persisted after accounting for the imbalance in group sizes (RRMS *n* = 13; SPMS *n* = 19). By contrast, dysphagia severity scores (DYMUS and EAT-10) and MUST scores did not differ significantly between MS subtypes (all *p* > 0.11). EDSS score was significantly correlated with calf circumference (ρ = 0.351, *p* = 0.049) but showed no significant associations with dysphagia severity, MUST score, or handgrip strength (all *p* > 0.50).

## 4. Discussion

This study is among the first to simultaneously and systematically evaluate dysphagia severity, nutritional status, and muscle function using validated, consensus-based instruments in ambulatory MS patients with confirmed dysphagia. The principal finding is that dysphagia severity—as measured by both the DYMUS and EAT-10—is significantly associated with malnutrition risk and GLIM-defined malnutrition, supporting H1. By contrast, no significant association was found between dysphagia severity and the primary EWGSOP2 muscle function parameters (handgrip strength and calf circumference), failing to support H2 in this sample.

The prevalence of GLIM-defined malnutrition in our sample (37.5%) is consistent with rates reported for neurological disease populations. Novotná et al., in validating the DYMUS questionnaire in MS, confirmed it as a reliable screening tool for swallowing impairment and reported that greater dysphagia severity is associated with higher disability [7]. Our finding that higher DYMUS scores were associated with greater odds of malnutrition (OR = 1.707; 95% CI: 1.138–2.560)—an association that remained after exploratory adjustment for EDSS—is consistent with, but cannot establish, a hypothesized pathway from dysphagia to reduced intake to disease-related malnutrition; this sequence requires longitudinal confirmation. This finding aligns with observations by Yun et al. and Baijens et al. who reported associations between dysphagia and malnutrition-related sarcopenia in neurological populations [6,22].

The strong correlation between dysphagia severity and MUST score, along with the significant MUST risk gradient across DYMUS severity categories, underscores the clinical relevance of integrating dysphagia and nutritional screening. Importantly, the GLIM framework used in this study explicitly incorporates dysphagia-induced reduced food intake as an aetiological criterion, making it conceptually coherent for this population and reinforcing the value of a combined screening-diagnosis approach as recommended by the GLIM consensus [11]. The agreement between MUST-based risk classification and GLIM-confirmed malnutrition further supports the utility of MUST as a pre-screening step in ambulatory MS clinics, consistent with findings in hospitalised populations [23]. It should be emphasised, however, that MUST and GLIM capture composite nutritional risk rather than confirmed protein–energy deficiency; accordingly, the present findings concern nutritional risk indicators and should not be interpreted as evidence of a verified nutritional deficit.

The absence of significant associations between dysphagia severity and handgrip strength or calf circumference (H2 not supported) requires careful consideration. Several explanations are plausible. First, in patients who still ambulate, compensatory mechanisms and residual physical activity may partially preserve muscle mass and strength independently of dietary adequacy. Second, the multifactorial aetiology of muscle weakness in MS—encompassing central motor drive impairment, spasticity, and disuse—may produce a dissociation between dysphagia-driven nutritional deficit and the predominantly neurological determinants of grip strength in this sample. Third, calf circumference, while a practical and GLIM-endorsed proxy for muscle mass in resource-limited settings, lacks the precision of dual-energy X-ray absorptiometry (DXA) or bioelectrical impedance analysis (BIA) and may be insufficiently sensitive to detect early or moderate reductions in lean mass [11]. Fourth, this study may have been underpowered to detect modest correlations: with N = 32, the detectable minimum effect size for a significant two-tailed Spearman correlation at α = 0.05, power = 0.80 is r ≈ 0.49, meaning weak-to-moderate correlations (r < 0.30) would be missed.

The finding that DYMUS and EAT-10 correlated moderately with continuous gait speed but not with categorical sarcopenia classification warrants separate discussion. The near-universal presence of low gait speed in this sample (93.8%) constitutes a substantial floor effect that renders the categorical EWGSOP2 gait speed cut-off (<0.8 m/s) of limited discriminative utility in this cohort. The continuous correlation may reflect a shared neurological substrate: brainstem and spinal cord lesions that impair swallowing can simultaneously impair coordinated limb movements and postural control, a parallel consistent with findings from DOSS-based MS studies showing that dysphagia correlates with functional disability [24]. This should not, however, be interpreted as evidence that dysphagia drives physical performance through a nutritional pathway in ambulatory patients.

The significantly lower handgrip strength in SPMS compared with RRMS (20.0 vs. 24.0 kg) is consistent with the progressive nature of SPMS and its associated neuromuscular involvement. Notably, dysphagia severity and nutritional risk did not differ between subtypes, suggesting that muscle function impairment in SPMS is predominantly neurological rather than nutritionally mediated. This dissociation between muscle function (MS-subtype dependent) and nutritional status (dysphagia-severity dependent) provides clinically important nuance: interventions targeting dysphagia and nutrition may be most relevant to malnutrition outcomes, while subtype-specific neuromuscular rehabilitation programmes may be required for muscle function. Because the cohort was enriched for SPMS (59.4%) and moderate disability (median EDSS 4.0), the observed muscle-function impairments are at least partly attributable to disease subtype and progression rather than to dysphagia or nutritional status.

The severe-sarcopenia rate (46.9%) cannot be validly interpreted in this sample, as the near-universal low gait speed (floor effect) drove the EWGSOP2 classification; accurate classification would require body composition measurement (DXA or BIA). With 93.8% of patients meeting the low gait speed criterion, virtually all patients with additionally low handgrip strength were automatically classified as severely sarcopenic, regardless of calf circumference. Future studies incorporating sensitive body composition measures (DXA or BIA) would enable more accurate sarcopenia classification in ambulatory MS populations.

### Strengths and Limitations

This study has several methodological strengths. A multidisciplinary battery of validated instruments was applied simultaneously, encompassing swallowing function, nutritional risk, malnutrition diagnosis, and muscle function within a coherent conceptual framework (GLIM + EWGSOP2). The use of MS-specific dysphagia instruments (DYMUS, EAT-10), a complementary bedside aspiration safety screen (Yale Swallow Protocol), and consensus-based malnutrition criteria (GLIM) reflects current best practice. The consecutive sampling strategy minimises selection bias within the study centre.

Several important limitations must be acknowledged. The cross-sectional design precludes any inference of causality between dysphagia, nutritional decline, and muscle function loss. The sample size (N = 32), though meeting the pre-specified threshold for the primary correlation analysis, limits the power to detect modest effect sizes and precludes robust multivariable modelling; the exploratory logistic regression (EPV = 6) should be interpreted accordingly. The study was conducted at a single centre, which may limit generalisability to broader MS populations with different disability profiles. Muscle mass was assessed by calf circumference rather than DXA or BIA, introducing measurement imprecision for the GLIM muscle mass phenotypic criterion and potentially correlated misclassification across the GLIM and EWGSOP2 frameworks, which share this single proxy. Because calf circumference is influenced by adiposity and oedema and may remain normal despite reduced lean tissue, particularly in this overweight cohort (median BMI 25.5 kg/m^2^), it may produce false-negative muscle-mass classification; the null associations between dysphagia severity and calf-dependent measures should therefore be interpreted as possibly underestimated rather than as confirmed absence of association. The near-universal presence of low gait speed (floor effect) substantially constrains the interpretability of physical performance as a sarcopenia indicator in this ambulatory cohort. Finally, the study did not capture actual dietary intake (energy and protein), diet composition, food-texture modifications, or use of oral or enteral nutritional support, nor aspiration pneumonia history, depression, fatigue, medication status, or laboratory markers of inflammation (C-reactive protein) and nutritional status (albumin, prealbumin). Consequently, the association between dysphagia severity and MUST/GLIM-defined status cannot establish whether patients had genuinely insufficient protein or energy intake, nor disentangle the contributions of inflammatory burden, disease activity, comorbidity, depression, fatigue, or medication effects from those of reduced intake; MUST and GLIM should therefore be read as composite nutritional-risk indicators rather than verified intake deficits. A further methodological consideration concerns the potential for partial structural overlap between the primary predictor (DYMUS score) and the GLIM outcome: the GLIM framework explicitly accepts dysphagia-related reduced food intake as an aetiological criterion. Consequently, patients with higher DYMUS scores may partially fulfil GLIM criteria on the basis of dysphagia itself rather than solely through its downstream nutritional consequences. While associations between DYMUS and the phenotypic GLIM criteria (unintentional weight loss, low BMI) are less directly affected by this concern, the logistic regression findings should be interpreted with this structural overlap in mind, and replication in studies using exclusively phenotypic GLIM outcomes would further reduce this concern. In a phenotype-only operationalization, 81.3% of the sample met ≥ 1 phenotypic criterion, leaving only six phenotype-negative patients and insufficient variability for informative modelling; its null estimate should therefore not be read as contradicting the primary finding. By contrast, re-deriving GLIM without the dysphagia-specific aetiologic criterion left the association unchanged, indicating that it is not solely an artefact of criterion overlap, though longitudinal data remain necessary to confirm directionality. Taken together, these sensitivity analyses reduce the likelihood that the observed association is a methodological artefact but do not completely eliminate the conceptual overlap between the predictor and the outcome; they should therefore be regarded as supportive and exploratory rather than as definitive evidence of an independent association. The disproportionate representation of SPMS patients (59.4%) is higher than typically reported in population-based MS cohorts and likely reflects referral to a tertiary neurological rehabilitation unit (referral/selection bias) and therefore over-represents secondary progressive disease relative to population-based MS cohorts. Consequently, the findings cannot be directly generalised to typical RRMS outpatients or to patients with lower disability, in whom dysphagia, nutritional risk, and muscle function may differ substantially. Additionally, the MUST medium-risk group contained only two patients (*n* = 2), rendering the Kruskal–Wallis gradient analysis across the three MUST categories unreliable; this finding should be interpreted with caution. Finally, no patients were classified as having “confirmed sarcopenia” (low muscle strength plus low muscle mass without low gait speed) in this cohort; this is attributable to the near-universal presence of low gait speed, which automatically elevated all patients with low handgrip strength and low calf circumference to the “severe” category under the EWGSOP2 algorithm.

## 5. Conclusions

In this small, single-centre, cross-sectional study of ambulatory MS patients with dysphagia, greater dysphagia severity was associated with higher nutritional risk and with GLIM-defined malnutrition, but not with the primary muscle strength and mass indicators examined. Because MUST and GLIM reflect composite nutritional risk rather than confirmed protein–energy deficiency, this association should be regarded as exploratory and hypothesis-generating and requires confirmation in larger prospective cohorts. The present data did not permit a reliable estimate of sarcopenia prevalence in MS patients with dysphagia, owing to the near-universal reduction in gait speed and the absence of body composition measurement; all sarcopenia-related findings should therefore be considered preliminary. As a preliminary practical consideration, and not as a consequence of an established causal relationship, these findings may support the combined use of standardised dysphagia and nutritional screening (e.g., DYMUS or EAT-10 alongside MUST and GLIM) in multidisciplinary MS outpatient care. Future longitudinal studies with adequate sample sizes, gold-standard body composition methodology, quantified dietary and protein intake, food-texture and nutritional-support data, and inflammatory/biochemical markers are needed to clarify the temporal relationship between dysphagia, nutritional decline, and muscle function loss in this population.

## Figures and Tables

**Table 1:** Participant characteristics (*n* = 32).

Characteristic	Total Sample (*n* = 32)	Reference/Range
**Sociodemographic**		
Age, years, mean ± SD	40.3 ± 8.4	22–60
Sex, female, *n* (%)	26 (81.2)	—
Marital status, married, *n* (%)	7 (21.9)	—
**Clinical Characteristics**		
MS type—RRMS, *n* (%)	13 (40.6)	—
MS type—SPMS, *n* (%)	19 (59.4)	—
Disease duration, years, median (IQR)	11.5 (7.0–15.0)	1–30+
Relapse count, last 2 years, median (IQR)	2.0 (1.0–2.0)	—
EDSS score, median (IQR)	4.0 (3.5–4.0)	2.0–5.5
**Anthropometry**		
Height, cm, mean ± SD	164.8 ± 8.7	—
Weight, kg, median (IQR)	68.5 (56.8–83.2)	—
BMI, kg/m^2^, median (IQR)	25.5 (21.1–31.1)	18.2–40.0
Calf circumference, cm, mean ± SD	32.5 ± 3.8	25.0–42.0
Unintentional weight loss (past 6 months), *n* (%)	17 (53.1)	—
**Swallowing Assessment**		
DYMUS score, median (IQR)	4.0 (3.0–6.2)	2–10
Mild dysphagia (DYMUS 1–3), *n* (%)	10 (31.2)	—
Moderate dysphagia (DYMUS 4–6), *n* (%)	14 (43.8)	—
Severe dysphagia (DYMUS 7–10), *n* (%)	8 (25.0)	—
EAT-10 score, median (IQR)	11.0 (5.0–23.5)	0–40
EAT-10 ≥ 3 (clinical dysphagia), *n* (%)	30 (93.8)	—
Yale Swallow Protocol positive, *n* (%)	5 (15.6)	—
**Nutritional Status**		
MUST score, median (IQR)	0.0 (0.0–3.0)	0–6
Low risk (MUST = 0), *n* (%)	19 (59.4)	—
Medium risk (MUST = 1), *n* (%)	2 (6.2)	—
High risk (MUST ≥ 2), *n* (%)	11 (34.4)	—
GLIM present, *n* (%)	12 (37.5)	—
GLIM severity—moderate, *n* (%)	3 (25.0) *	—
GLIM severity—severe, *n* (%)	9 (75.0) *	—
**Muscle Function (EWGSOP2)**		
Handgrip strength, kg, median (IQR)	20.5 (19.0–24.0)	17–33
Low muscle strength, *n* (%)	23 (71.9)	—
Low calf circumference (<33 cm), *n* (%)	18 (56.2)	—
4 m gait speed, m/s, median (IQR)	0.69 (0.60–0.80)	0.50–1.00
Low gait speed (<0.8 m/s), *n* (%)	30 (93.8)	—
No sarcopenia, *n* (%)	9 (28.1)	—
Possible sarcopenia, *n* (%)	8 (25.0)	—
Severe sarcopenia, *n* (%)	15 (46.9)	—

BMI: body mass index; DYMUS: Dysphagia in Multiple Sclerosis questionnaire; EAT-10: Eating Assessment Tool-10; EDSS: Expanded Disability Status Scale; EWGSOP2: European Working Group on Sarcopenia in Older People 2; GLIM: Global Leadership Initiative on Malnutrition; IQR: interquartile range; MUST: Malnutrition Universal Screening Tool; RRMS: relapsing-remitting multiple sclerosis; SD: standard deviation; SPMS: secondary progressive multiple sclerosis. —: not applicable (no established reference range for the given variable). * Percentages for GLIM severity are calculated among malnourished patients only (*n* = 12). Because 93.8% of patients met the low-gait speed criterion, patients with concurrent low handgrip strength and low calf circumference were automatically classified as severely sarcopenic under the EWGSOP2 algorithm; the severe-sarcopenia rate (46.9%) is therefore likely overestimated and the categorisation should be interpreted as non-informative in this cohort.

**Table 2:** Spearman’s correlations between dysphagia severity and nutritional status indices.

Association	Spearman ρ	*p*-Value	Interpretation
DYMUS score–MUST score	0.596	<0.001 *	Moderate–strong
EAT-10 score–MUST score	0.518	0.002 *	Moderate–strong
DYMUS score–BMI	−0.241	0.185	Weak (ns)
EAT-10 score–BMI	−0.337	0.059	Weak–moderate (ns)
DYMUS score–Calf circumference	0.170	0.353	Weak (ns)
EAT-10 score–Calf circumference	0.036	0.847	Negligible (ns)

ns: not statistically significant; *: *p* < 0.05. Calf circumference appears in both Table 2 and Table 4 because a single standardised measurement serves as the muscle-mass proxy in both the GLIM and EWGSOP2 frameworks; the two entries therefore represent the same association reported once under each framework, not two independent findings. DYMUS: Dysphagia in Multiple Sclerosis Questionnaire; EAT-10: Eating Assessment Tool-10; MUST: Malnutrition Universal Screening Tool; BMI: body mass index.

**Table 3:** Comparison of dysphagia and nutritional indicators according to GLIM-defined malnutrition status.

Variable	Malnutrition Present (*n* = 12)	Malnutrition Absent (*n* = 20)	U	*p*
DYMUS score	6.5 (5.5–10.0)	4.0 (3.0–5.0)	184	0.012 *
EAT-10 score	18.0 (10.5–36.5)	11.0 (4.8–13.5)	172	0.042 *
MUST score	4.0 (2.8–5.0)	0.0 (0.0–0.0)	240	<0.001 *
BMI (kg/m^2^)	20.8 (19.1–22.0)	27.2 (25.0–34.6)	32	<0.001 *
Calf circumference (cm)	32.0 (30.0–32.2)	33.5 (30.8–35.0)	80	0.126

Values are presented as median [interquartile range]. Between-group comparisons were performed using the Mann–Whitney U test. *: *p* < 0.05. BMI: body mass index; DYMUS: Dysphagia in Multiple Sclerosis Questionnaire; EAT-10: Eating Assessment Tool-10; GLIM: Global Leadership Initiative on Malnutrition; MUST: Malnutrition Universal Screening Tool.

**Table 4:** Spearman correlations between dysphagia severity and muscle strength/performance indicators.

Association	Spearman ρ	*p*-Value	Interpretation
DYMUS score–Handgrip strength (kg)	0.066	0.720	Negligible (ns)
EAT-10 score–Handgrip strength (kg)	−0.021	0.908	Negligible (ns)
DYMUS score–Calf circumference (cm)	0.170	0.353	Weak (ns)
EAT-10 score–Calf circumference (cm)	0.036	0.847	Negligible (ns)
DYMUS score–4 m gait speed (m/s) †	−0.495	0.004 *	Moderate (exploratory)
EAT-10 score–4 m gait speed (m/s) †	−0.525	0.002 *	Moderate–strong (exploratory)

ns: not statistically significant; *: *p* < 0.05. † 4 m gait speed analysed as a continuous variable (m/s) due to a pronounced floor effect in the categorical classification (30/32 patients classified as low gait speed). Results should be interpreted as exploratory.

**Table 5:** Exploratory binary logistic regression: predictors of GLIM-defined malnutrition (dependent variable: malnutrition present = 1).

Predictor	OR	95% CI	*p*-Value
**Model 1: Unadjusted**			
DYMUS score	1.707	1.138–2.560	0.010 *
**Model 2: Adjusted for EDSS (exploratory, EPV = 6)**			
DYMUS score	1.722	1.144–2.591	0.009 *
EDSS score	1.268	0.427–3.769	0.669

CI: confidence interval; EDSS: Expanded Disability Status Scale; EPV: events per variable (12 events); OR: odds ratio. *: *p* < 0.05.

## Data Availability

The datasets generated and/or analysed during the current study are not publicly available due to institutional data protection policies but are available from the corresponding author on reasonable request.

## Abbreviations

The following abbreviations are used in this manuscript:

DYMUS	Dysphagia in Multiple Sclerosis
EAT-10	Eating Assessment Tool-10
EWGSOP2	European Working Group on Sarcopenia in Older People 2
GLIM	Global Leadership Initiative on Malnutrition
MS	Multiple Sclerosis
MUST	Malnutrition Universal Screening Tool
RRMS	Relapsing-Remitting Multiple Sclerosis
SPMS	Secondary Progressive Multiple Sclerosis
YSP	Yale Swallow Protocol
